# Supplementary material for: Genotype-environment interaction and the maintenance of genetic variation: an empirical study of Lobelia inflata (Campanulaceae)
Source: R Soc Open Sci. 2020 Mar 18;7(3):191720. doi: 10.1098/rsos.191720 (PMC7137973; doi:10.1098/rsos.191720)
Supplement: Tables S1 and S2 [file rsos191720supp1.docx]

**Supplementary Material:**

Table S1. The number of replicates of the sixteen, four-locus microsatellite genotypes present in each of the four environments used in the study. Genotypes without sufficient replication in a particular environment (–) were not used in analyses that included that environment. A shared letter within each of the four loci (but not among loci) indicates a shared microsatellite allele. The four loci used correspond with Linflata8, Linflata14, Linflata15 and Linflata21 from [1, 2].

| Genotype | Field | Long | Short | Garden |
| --- | --- | --- | --- | --- |
| AABD | – | 8 | 6 | 6 |
| ACBA | 10 | 5 | 6 | 3 |
| ACEA | 2 | 7 | 2 | – |
| ACED | 2 | 6 | 4 | 4 |
| CAAA | 2 | 10 | 9 | – |
| CABA | – | 5 | 4 | – |
| CAEA | 3 | 5 | 5 | 3 |
| CCAA | 8 | 16 | 14 | 14 |
| CCAD | 2 | 5 | 7 | – |
| CCBD | 3 | 14 | 11 | – |
| CCEA | 2 | 7 | 6 | 3 |
| CEDA | 2 | 7 | 6 | – |
| DABA | – | 7 | 6 | – |
| DAEA | – | 8 | 5 | 4 |
| DCAD | – | 14 | 13 | – |
| DCBA | 3 | 13 | 10 | 7 |

Table S2. Allele frequencies at each of the four microsatellite loci at the time of field collection for the 16 unique genotypes used in the study. The use of the same letter names across the four loci is for convenience and does not imply the same allele.

| Allele | Locus 1 | Locus 2 | Locus 3 | Locus 4 |
| --- | --- | --- | --- | --- |
| A | 0.341 | 0.205 | 0.295 | 0.795 |
| B | 0.000 | 0.000 | 0.432 | 0.000 |
| C | 0.523 | 0.750 | 0.000 | 0.000 |
| D | 0.136 | 0.000 | 0.045 | 0.205 |
| E | 0.000 | 0.045 | 0.227 | 0.000 |

[1] Hughes, P.W., Jaworski, A.F., Davis, C.S., Aitken, S.M. & Simons, A.M. 2014 Development of Polymorphic Microsatellite Markers for Indian Tobacco,Lobelia inflata(Campanulaceae). *Applications in Plant Sciences* **2**, 1300096. (doi:10.3732/apps.1300096).

[2] Hughes, P.W. & Simons, A.M. 2015 Microsatellite evidence for obligate autogamy, but abundant genetic variation in the herbaceous monocarp Lobelia inflata (Campanulaceae). *J Evol Biol* **28**, 2068-2077. (doi:10.1111/jeb.12734).
